# Hospital-diagnosed infections with *Escherichia coli* clonal group ST131 are mostly acquired in the community

**DOI:** 10.1038/s41598-021-85116-6

**Published:** 2021-03-11

**Authors:** Allison Muller, Houssein Gbaguidi-Haore, Pascal Cholley, Didier Hocquet, Marlène Sauget, Xavier Bertrand

**Affiliations:** 1 Hygiène Hospitalière, Centre Hospitalier Universitaire, 3 Boulevard Fleming, 25030 Besançon, Cedex, France; 2 UMR CNRS 6249 Chrono-Environnement, Université de Bourgogne-Franche-Comté, Besançon, France; 3 Centre de Ressources Biologiques - Filière Microbiologique de Besançon, Centre Hospitalier Universitaire, Besançon, France

**Keywords:** Infectious-disease epidemiology, Bacterial pathogenesis

## Abstract

The worldwide spread of *E. coli* ST131 has significantly contributed to the dissemination of *E. coli* producing extended-spectrum β-lactamases (ESBL). In a French University hospital, we assessed the molecular features of ESBL-producing *E. coli* and identified risk factors in patients for colonization or infection with *E. coli* ST131. Over a 2-year period (2015–2017), each patient with at least one clinical isolate or one screening isolate positive with ESBL-producing *E. coli* were included (n = 491). The ST131 clonal group accounted for 17.5% (n = 86) of all ESBL-producing *E. coli* and represented 57.3% isolates of phylogroup B2. *FimH*-based sub-typing showed that 79.1% (68/86) of ST131 isolates were *fimH30,* among which 67.6% (n = 46), 20.6% (n = 14) and 11.8% (n = 8) isolates harbored genes encoding the ESBL CTX-M-15, CTX-M-27, and CTX-M-14, respectively. The multivariate analysis identified two factors independently associated with ST131 ESBL-producing *E. coli* isolates: infection (Odds ratio [OR] = 1.887, 95% confidence interval [CI]: 1.143–3.115; p = 0.013) and community acquisition (OR = 2.220, 95% CI: 1.335–3.693; p = 0.002). In conclusion, our study confirmed the predominance of ST131 clonal group among ESBL-producing *E. coli* and the difficulty to identify common risk factors associated with carriage of this pandemic clonal group.

## Introduction

The worldwide spread of Sequence Type 131 (ST131) *Escherichia coli* has significantly contributed to the dissemination of *E. coli* resistant to fluoroquinolones (FQ) and producing extended-spectrum β-lactamases (ESBL)^1^. The molecular epidemiology of BLSE-encoding gene in *E. coli* is a typical model to decipher the spread of antibiotic resistance in pathogenic bacteria. Hence, it combines two levels of complexity: a first level related to the strain tested through the examination of core genes and the second level related the *bla*_ESBL_ genes carried by the accessory genome and mobile genetic elements. These isolates exhibit the usual virulence factors associated with extra-intestinal pathogenic *E. coli* belonging to phylogroup B2^2^. Recent analyses of the whole genome sequences of large collections of ST131 established that multi-drug-resistant clades C1/*fimH30*-R and C2/*fimH30*-Rx emerged in the 1980′s, most likely in North America^3,4^. The evolutionary history suggests that *fimH30* allele was acquired by a single FQ-susceptible ancestor, which later became resistant to this antibiotic family (subclone *H*30R). The further acquisition of *bla*_CTX-M-15_ created another distinct subclone (*H*30-Rx) which derived from a single common ancestor within *H*30R. *FimH30* subclones have disseminated globally as a result of clonal expansion. Despite these advances in our knowledge of the epidemiology of ST131, there remain considerable gaps regarding reservoirs, mechanisms of transmission and patient risk factors of ST131 acquisition. In a previous study, we described the long-term epidemiology of this clonal group in our University hospital, and demonstrated that it was predominant, although it only accounts for less than 20% of ESBL-producing *E. coli*^5^. The objectives of this study were to assess the molecular features of ESBL-producing *E. coli* and to identify risk factors for colonization or infection with the clone ST131.

## Results

Over the two-year survey, 505 non-duplicate ESBL-producing *E. coli* were isolated from cultures from eligible patients. Among these, 491 (97.2%) isolates were available for further analysis. Overall, ESBL-producing *E. coli* were isolated from urines samples (n = 208, 42.4%), rectal swab or stools (n = 150, 30.5%), blood cultures (n = 49, 10.0%) and other samples (n = 84, 17.1%).

### Characterization of the ESBL-producing *E. coli* isolates

The B2 phylogroup and clonal group ST131 accounted for 30.5% (n = 150) and 17.5% (n = 86) of all ESBL-producing *E. coli*, respectively. Clonal group ST131 represented 57.3% isolates of phylogroup B2 with 83 ST131, 1 ST2268, 1 ST2508 and 1 ST3565 (Table 1). Regarding the other ESBL-producing *E. coli*, B2 non-ST131 (n = 64) belonged to 29 different STs. The most common ST after ST131 was ST141 (n = 13) followed by ST12 (n = 7), ST95 (n = 6) and ST1722 (n = 4) (Table 1). Among the 86 clonal group ST131, 68 isolates (79.1%) were *fimH30* and among them 46 (67.6%), 14 (20.6%) and 8 (11.8%) isolates harbored the genes *bla*_CTX-M-15_, *bla*_CTX-M-27_, and *bla*_CTX-M-14_, respectively (Table 1). Among B2 non-ST131, 29 isolates (45.3%), 12 (18.8%) and 11 (17.2%) harbored the genes *bla*_CTX-M-14_, *bla*_CTX-M-1_, and *bla*_CTX-M-15_, respectively (Table 1). Among the 65 ST131 *fimH30* isolates, the 44 isolates that produced CTX-M-15 belonged to subclade C2, and the 13 and 8 isolates that produced CTX-M-27 and CTX-M-14, respectively, belong to subclade C1 (Table 1).

**Table 1:** Sequence Type distribution for 150 ESBL-producing clinical *E. coli* belonging to the phylogroup B2 in the University hospital of Besançon (France) between February 2015 and January 2017.

Clonal complex (No. of isolates)	Sequence type (no. of isolates)	*fimH* type (no. of isolates)	ESBL gene (no. of isolates)
CC131 (86)	ST131 (83)	H30 (65)	*bla*_CTX-M-15_ (44), *bla*_CTX-M-27_ (13) and *bla*_CTX-M-14_ (8)
		H41 (9)	*bla*_CTX-M-15_ (3), *bla*_CTX-M-14_ (2), *bla*_CTX-M-27_ (2), *bla*_CTX-M-1_ (1) and *bla*_CTX-M-55_ (1)
		H52 (2)	*bla*_CTX-M-1_ (2)
		H54 (5)	*bla*_CTX-M-9_ (3), *bla*_CTX-M-15_ (1), and *bla*_CTX-M-14_ (1)
		H141 (1)	*bla*_CTX-M-1_
		H153 (1)	*bla*_CTX-M-27_
	ST2268 (1)	H30	*bla*_CTX-M-27_
	ST2508 (1)	H30	*bla*_CTX-M-15_
	ST3565 (1)	H30	*bla*_CTX-M-15_
Non-CC131 (64)	ST141 (13)	–	*bla*_CTX-M-14_ (7), *bla*_CTX-M-15_ (2), *bla*_CTX-M-3_ (2), *bla*_CTX-M-65_ (1), and *bla*_TEM-like_ (1)
	ST12 (7)	–	*bla*_CTX-M-14_ (5) and *bla*_CTX-M-1_ (2)
	ST95 (6)	–	*bla*_CTX-M-14_ (4) and *bla*_CTX-M-15_ (2)
	ST1722 (4)	-	*bla*_CTX-M-15_ (2), *bla*_CTX-M-14_ (1) and *bla*_CTX-M-27_ (1)
	ST127 (3), ST4508 (3), ST349 (2), ST 457 (2), ST73 (2), ST 998 (2), ST1154 (1), ST1485(1), ST2372 (1), ST2914(1), ST3500 (1), ST357 (1), ST404 (1), ST491 (1), ST538 (1), ST569 (1), ST6161 (1), ST638 (1), ST681 (1), ST999 (1), new STs (6)	–	*bla*_CTX-M-14_ (12), *bla*_CTX-M-1_ (10), *bla*_CTX-M-15_ (5), *bla*_CTX-M-3_ (1), *bla*_CTX-M-9_ (1), *bla*_SHV_ (3), *bla*_SHV2a_ (1) and *bla*_TEM_ (1)

### Risk factors associated with ST131 ESBL-producing E. coli

Univariate analysis revealed that, accommodation in a nursing home (p = 0.002) and hospitalization in a medical unit (p = 0.001) were significantly associated with the carriage of ST131 isolates (Table 2). In contrast, solid cancer/blood cancer, immunosuppression, home housing, hospitalization in intensive care and hematology units were protective factors (*p* < 0.05). ST131 *E. coli* isolates were more frequently associated with an infection than colonization (defined by the presence of clinical signs or healthy carriage, respectively) (p < 0.001). In addition, they were more frequently isolated from urinary samples (p = 0.005) and community acquired (p < 0.001). Rectal/stool samples were associated with non-ST131 *E. coli* (p < 0.001). Time between admission (p < 0.001) and sample collection and length of stay were shorter for patients with ST131 isolates (p = 0.018). Moreover, for these patients 30-day all-cause mortality was lower (p = 0.004). Multivariate analysis identified only two factors positively associated with ST131 ESBL-producing *E. coli* isolates: infection (*p* = 0.013) and community acquisition (p = 0.002) (Table 3).

**Table 2:** Review of risk factors associated with *Escherichia coli* ST131 carriage.

Country (reference)	Period and setting	Samples, *E. coli* isolate type	Design and number of isolates	Risk factors associated to ST131 Univariate analysis	Risk factors associated to ST131 multivariate analysis
Taiwan^18^	2005–2010 Hospital	Bacteremia (blood culture), ESBL producers	ST131 *vs.* non-ST131 122 (36/86)	UTI	Absence of urinary catheter, secondary bacteremia
France^19^	2008–2009 Hospital	Clinical samples, CTX-M-producers	ST131 *vs.* non-ST131 152 (55/97)	Elderly, collective housing, functionally dependent before hospitalization, LTCF residents, urinary tract infection	LTCF
Spain^20^	2010 Hospital and community	Clinical samples, ESBL producers and non- producers	ESBL ST131 *vs.* ESBL non-ST131 146 (34/112)	Community acquisition	
			Non-ESBL ST131 *vs.* non-ESBL non-ST131 398 (110/288)	Elderly, Diabetes mellitus, exposure to antibiotics	Diabetes mellitus, antibiotic exposure
Spain^21^	2010–2012 Hospital	Bacteremia (blood culture), non-ESBL producers	025b:H4-ST131 *vs.* non 025b:H4-ST131 89 (33/56)	Recent surgery	Recent surgery, unknown source of bacteremia
USA^22^	2011 Hospital	Clinical samples, ESBL producers and non- producers	ST131 *vs.* non-ST131 299 (80/219)	Elderly, LTCF residents, hypertension, genitourinary anomalies, cancer, recent surgery, recurrent UTI, antibiotic exposure, healthcare associated	Elderly, LCTF, UTI
Spain^23^	2012–2013 Community (household members)	Rectal colonization (swabs), ESBL producers and non- producers	ST131 *vs.* non-ST131 64 (18/46)	Elderly, dependent for basic activities, not sharing bathroom with index case, antibiotic exposure, PPI use	PPI use, elderly
	Hospital (contact patients)		ST 131 *vs.* non-ST131 54 (10/44)	Elderly, dependent for basic activities, bed-ridden, urinary catheter	Dependent for activities, urinary catheter
USA^24^	2012–2013 US veteran affairs	Clinical samples, ESBL producers and non- producers	ST131 *vs.* non-ST131 469 (66/403)	Medical lines, hospital stay, nursing home stay, surgery, exposure to antibiotics	
Korea^25^	2013–2014 Hospital	Bacteremia (blood culture), ESBL producers	ST131 *vs.* non-ST131 124 (58/66)	Diabetes mellitus , chronic renal insufficiency, absence of active cancer, absence of prior chemotherapy history, absence of surgery history	Diabetes mellitus, absence of prior chemotherapy
Korea^26^	2016–2017 Community	Female genital tract sample, ESBL producers and non- producers	ST 131 *vs.* non-ST131 91 (20/71)	None	
Taiwan^27^	2017 Community (Healthy patients)	Fecal samples, ESBL producers and non- producers	ST131 *vs.* non-ST131 724(22/702)	Stroke, cancer	Stroke, cancer
France (this study)	2015–2017 Hospital	Clinical and screening samples, ESBL producers	ST131 *vs.* non-ST131 491 (83/408)	Nursing home residency, medicine hospitalization, infection, urinary infection, community acquisition, time between admission and sample, hospitalization length	Infection, community acquisition

*ESBL* extended-spectrum-*β*-lactamase-producing; *BSI* bloodstream infection; *UTI* urinary tract infection; *LTCF* long-term care facility, *PPI* proton pump inhibitors.

**Table 3:** Multivariate analysis of risk factors associated with CC131 ESBL-producing *E. coli* clinical isolates using logistic regression.

Variable	Odds ratio	95% confidence interval	P value
Female gender	1.328	0.806–2.186	0.266
Solid cancer/blood cancer	0.586	0.306–1.120	0.106
Immunosuppression	0.651	0.311–1.360	0.253
Nursing home residents	2.098	0.811–5.429	0.127
Intensive care hospitalization	0.699	0.347–1.406	0.315
Infection	1.887	1.143–3.115	0.013
Community acquisition	2.220	1.335–3.693	0.002

## Discussion

So far, ten studies on risk factors associated with ST131 have been reported worldwide. The main results of these studies are summarized in Table 2. They identified many risk factors for acquisition of ST131: elderly, long-term-residency at care facility or nursing homes, diabetes mellitus, absence of urinary catheter, previous exposure to antibiotics or absence of prior antibiotic treatment, recent surgery, use of proton pump inhibitor, or cancer. Risk factors were not consistent from one study to another, presumably due to variation in study designs, patient populations, or bacterial populations considered (i.e. all ST131 isolates or only ESBL producers). It may also reflect the variability of ST131 prevalence according to countries or populations. In our study, multivariate analysis associated ST131 isolates with infection, certainly reflecting the higher virulence of the phylogroup B2 and in line with the fact that ST131 infection are more frequently acquired in the community^1^. Overall, identification within the human hosts of risk factors associated with acquisition of ESBL-producing ST131 is not that simple. Our interpretation is that the global spread of ST131 relies more on bacterial factors than on host characteristics. A recent outstanding study have demonstrated that the success of ST131 is related to the ability of ST131 subclades to evolve toward separate ecological niches and to select genes involved in anaerobic metabolism and human colonization^6^. This adaptation certainly accounts for the long-term intestinal colonization and high transmissibility rate in both the community and hospital settings of *E. coli* ST131^7^.

The present study confirms previous data on ST131 epidemiology in our hospital^5^. First, the clone ST131 accounted for 17.5% but predominated among ESBL-producing *E. coli.* Secondly, the *fimH* typing revealed that 68 of the 86 clonal complex CC131 isolates (79.1%) were of *H*30Rx subclone and that pandemic clusters C2-M15 (n = 44; producing CTX-M-15) and C1-M27 (n = 13; producing CTX-M-27) were over-represented in the *H*30Rx subclone. *H*41 isolates (n = 9), very likely belonging to O16 serotype clade A, represented the second most frequent *fimH* type. *H*22 clade B (the predecessor of *fimH30*) was not detected although present in 2010–2012 in our hospital^5^. Thirdly, ESBL-producing ST131 should be considered as a cluster of distinct clonal subsets with specific characteristics rather than as a unified entity.

Phylogroup B2 accounts for a large subset of EXPEC strains that are responsible for the majority of human extra-intestinal infections globally^2^. Until recently, ST131 remained the only phylogroup B2 clone that successfully evolved under antibiotic resistance by acquiring chromosomal resistance to fluoroquinolones and plasmids harboring *bla*_CTX-M_ genes^1^. Unexpectedly, more than 40% of our collection of ESBL-producing phylogroup B2 isolates were not ST131. We found historical clones responsible for UTIs (ST73, ST95) as well as emerging clones (ST127, ST141). Birgy and colleagues also recently described the presence of ST73 and ST95 in a large collection of ESBL-producing *E. coli* isolated from UTIs in French children^8^. ST141, which was the most frequent clone after ST131 in our collection of phylogroup B2 ESBL-producing *E. coli*, was considered as a Shiga toxin-producing *E. coli* (STEC) of serotype O2:H6^9^. All *E. coli* ST141-B2 isolates (n = 13) have already been tested in another study focusing on ST141 clonal group (manuscript in preparation). The sequencing of their full genomes revealed the absence of *stx* genes. More recently, an additional study suggests that ST141 isolates may be a hybrid positioned between Shiga toxin-producing and uropathogenic *E. coli*, serving as a “melting pot” for pathogroup conversion^10^. The emergence of ESBL in the clone ST141, as observed in our study, is worrisome and the investigation of phylogeny and the identification of virulence and resistance determinants are ongoing.

This study has some limitations. It would have been interesting to include all *E. coli* isolates, independently of the antibiotic resistance profile. However, ESBL production in *E. coli* is the main concern of public health and this justified the design of our study^11^. Although our study lacks novelty, our data are valuable since produced from a large cohort of more than 500 hospitalized patients over 2 years, with a systematic inclusion of patients with at least one clinical or screening positive *E. coli* isolate producing ESBL. This differentiates our study from previous ones. To conclude, our study confirms both the predominance of ST131 among ESBL-producing *E. coli* and the difficulty to identify common risk factors associated with this pandemic clonal group.

## Methods

### Setting, study period and patients included

We conducted a prospective observational cohort study for a 2-year period (February 2015–January 2017) in the Besançon University hospital, a 1200-bed hospital with approximately 50,000 admissions and 320,000 patient-days annually. Over the study period, all patients admitted in our hospital with at least one clinical isolate or one screening isolate positive with ESBL-producing *E. coli* were included (with the exception of consultations and day-care).

### Bacterial isolates

All the isolates were identified as *E. coli* by MALDI-TOF MS Microflex LT (Bruker Daltonik GmbH, Bremen, Germany) according to the manufacturer's recommendations and routinely tested for ESBL production using the synergy test^12^. The prevalence of *E. coli* BLSE in clinical isolates was 7.5% among total *E. coli.* All ESBL-producing *E. coli* isolates were stored at the Centre de Ressources Biologiques Filière Microbiologique, Besançon (CRB-FMB, Biobanque BB-0033-00090). We retained only the first isolate of ESBL-producing *E. coli* for patients with multiple samples positive with this species.

### Microbiological analysis

*E. coli* isolates were typed by phylogrouping as previously described^13^ and by MLST according to Achtman scheme for phylogroup B2 isolates^14^. ESBL-encoding genes (*bla*_CTX-M group 1_, *bla*_CTX-M group 9,_ *bla*_CTX-M group 2,_ *bla*_SHV_ and *bla*_TEM_,) were identified by PCR and sequencing^15^. For ST131 isolates, *fimH* sub-typing was determined by PCR and sequencing as previously described^16^. Among them, the distribution of the *fimH30* isolates into sub-clades C1 and C2 was further determined by multiplex PCR^17^.

### Patient data collection

Data were collected prospectively from patient medical records. We collected the following data for each patient: (i) demographical data (age, sex, housing), (ii) comorbidities and medical data (i.e. diabetes, immunodeficiency, cancer, high blood pressure, renal failure, liver failure, organ transplantation), (iii) medical history in the preceding year (hospitalizations, surgical interventions, antibiotic treatments), (iv) data about the current hospitalization (dates of admission and discharge, hospitalization ward, surgery, antibiotic treatment), (v) date and type of sample positive with ESBL-producing *E. coli*, (vi) data about the colonization or infection with ESBL-producing *E. coli* (type, site, origin) and (vii) patient outcome (length of stay, mortality at 30 days). We classified infections due to ESBL-producing *E. coli* as either community- or hospital-acquired when the first positive sample culture was collected ≤ 2 days or > 2 days after admission, respectively.

### Data analysis

All analyses were conducted using the software package Stata, version 14.1 (Stata Corp., College Station, TX, USA). Data are presented as means (standard deviations) for continuous variables and frequencies (percentages) for categorical variables. The comparison of continuous data was performed with the Student’s t-test or the Mann–Whitney U test while the categorical data were compared using the Pearson’s Chi-square test or Fisher’s exact test, as appropriate. Univariable and multivariable logistic regression analyses were carried out for determining factors associated with isolation of *E. coli* ST131 compared with *E. coli* non-ST131 in inpatients. Only variables with a *p* value ≤ 0.10 in univariable analysis were considered for multivariable analysis. A *p* value ≤ 0.05 was considered as significant.

### Ethics statement

This study has been performed in accordance with the Declaration of Helsinki. It has been approved by the ethics committee Comité de Protection des Personnes—Ouest III under the number 171267 prior to study commencement. The study has been declared under clinical trial number NCT02853708. A declaration to the CNIL (Commission nationale de l'informatique et des libertés) has been made under the number 1924594. Informed consent was obtained from the patients for the use of their clinical samples and data for this study. The patients whose anonymized data (age; risk factors) were given the following information: “Use of samples and microorganisms for research purposes: samples (blood samples, biopsies, surgical specimens) can be taken to establish a diagnosis and to adapt your treatment or that of your child. Some of these samples or the microorganisms they contain may be stored for diagnostic or research purposes. Your samples are anonymized. The medical data associated with the samples and the microorganisms are collected in a computer file authorized by the CNIL. You have the right to access and rectify the data entered. You may at any time reconsider your decision without any consequences for your care (or that of your child) and oppose the use of biopsies and operating documents for research by contacting the Franche-Comté Regional Tumorotheque (Tel. + 33 3 81 66 89 66) or oppose the use of blood samples or microorganisms for research by contacting the Centre de Ressource Biologique – Filière Microbiologique (Tel. + 33 3 70 63 21 34).” For the experiment, we applied the Orion guidelines (Debouck, F. Orion method: simple and effective systemic analysis of clinical events and precursors occurring in hospital medical practice. Elsevier Masson—March 2012).
